# Using Item Response Theory to Analyze the Relationship Between Health-Related Quality of Life and Health Risk Factors

**Published:** 2008-12-15

**Authors:** Yongwen Jiang, Jana Earl Hesser

**Affiliations:** Center for Health Data and Analysis, Rhode Island Department of Health; Rhode Island Department of Health, Providence, Rhode Island

## Abstract

Many researchers have presented results of the relationships between health-related quality of life (HRQOL) indicators (outcomes) and health risk factors using either linear or logistic regression modeling. We combined the results of multiple HRQOL models by using item response theory (IRT) to assess the association between multiple correlated HRQOL indicators and multiple demographic and health risk variables as predictors. The data source for the study was Rhode Island's 2004 Behavioral Risk Factor Surveillance System, which had a sample of 3,999 adults aged 18 years or older. We developed a single model for overall HRQOL by using IRT to assess the association between HRQOL indicators and multiple demographic and health risk variables as predictors. The strongest predictors for overall poor HRQOL were lower income, inability to work, unemployment, smoking, lack of exercise, asthma, obesity, and disability. IRT may serve as a solution for modeling multiple correlated outcomes in epidemiology. Application of IRT to epidemiologic data can help identify at-risk subgroups for targeted interventions.

## Introduction

The analysis of multiple correlated outcomes is relevant for epidemiologic research. Subjects in epidemiologic studies are often assessed using various outcomes measures. How can multiple correlated outcomes be used to establish an overall assessment of health risk? How can such a risk assessment be related to predictors? We used item response theory (IRT) to explore these questions and to build on our prior work with the health-related quality of life (HRQOL) indicators included in the Behavioral Risk Factor Surveillance System (BRFSS) (1).

HRQOL is a latent variable or latent trait that cannot be observed directly by a single measurement. A set of indicators (outcomes) (Figure) used to measure HRQOL is included in the BRFSS (2). Many researchers have examined the relationships between specific BRFSS HRQOL indicators and various health risk factors. Most of their studies have analyzed BRFSS HRQOL indicators by using either a logistic (3-11) or linear regression model (12). They are multivariable analyses that use multiple risk factor variables to predict specific HRQOL outcomes (eg, depression, activity limitation). However, individual HRQOL indicators are correlated because each HRQOL indicator measures a certain aspect of HRQOL. We found considerable overlap in results of the multiple single outcome models we described in our prior work (1). This finding led us to seek a single model that would combine results of the multiple HRQOL models. Item response theory (IRT) provided a possible means of accomplishing this objective because it enables analysis of multiple correlated outcomes within a single model. In this study, we apply IRT to Rhode Island's 2004 BRFSS data, which include 9 HRQOL indicators, to develop a single model for HRQOL.

**Figure .**
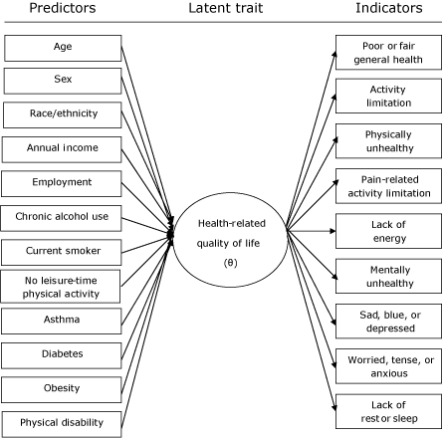
Item response theory model for the latent trait health-related quality of life (θ) with predictors and indicators.

IRT is popular in the fields of educational measurement and psychometrics. The method uses responses to a set of discrete items (indicators) (13) to estimate latent traits or latent variables that cannot be measured directly. For example, in educational testing, students' ability (latent trait) is estimated through their answers to multiple test items (indicators) (13). Our objective in applying the IRT model was to assess the effect of each of a number of predictors on overall HRQOL (latent variable).

## Methods

### Data source

We used data from Rhode Island's 2004 BRFSS for this analysis. From January through December 2004, the Rhode Island BRFSS conducted approximately 333 random-digit–dialed telephone interviews each month with adults aged 18 years or older, for a total of 3,999 during the calendar year. The response rate was 51%. Technical details of Rhode Island's 2004 BRFSS and Rhode Island's BRFSS data are available on request from the Center for Health Data and Analysis, Rhode Island Department of Health (14).

### Variables

Our study used the following 9 HRQOL questions from the 2004 Rhode Island BRFSS: 1) self-rated general health status; and self-reported number of healthy and unhealthy days in the previous 30 days for 2) physical health, 3) mental health, 4) physical or mental health-related activity limitation, 5) pain-related activity limitation, 6) sad, blue, or depressed, 7) worried, tense, or anxious, 8) lack of rest or sleep, and 9) lack of energy (1,2,15). We created 9 dichotomous indicator variables. The responses to the self-rated general health status question were dichotomized into "poor" (poor or fair) health or "good" (good, very good, or excellent) health. The indicators measured in days were dichotomized at a cutoff value of 14 or more days of poor health in the previous month compared to less than 14 days (3). We selected the 14-day minimum period because most of the publications we reviewed that use the BRFSS HRQOL indicators (outcomes) use the cutoff of 14 or more days compared to 13 or fewer days (3-5,7-11,16,17). Adopting this precedent ensured comparability. In addition, clinicians and clinical researchers often use this period as a marker for clinical depression and anxiety disorders, and long symptomatic durations are associated with high levels of activity limitation (2,18). Detailed definitions of the 9 indicators are available in our previous article (1) or are accessible through the Centers for Disease Control and Prevention's HRQOL Web site (2).

We chose 12 predictors for the analysis: 5 standard demographic measures (age, sex, race/Hispanic ethnicity, annual income, and employment status); 4 health conditions (asthma, diabetes, obesity, and physical disability); and 3 health risk behaviors (smoking, chronic alcohol use, and no leisure-time physical activity). These predictors paralleled the results of other studies that have examined relationships between a specific HRQOL indicator and various predictors (17,19), or that have examined multiple HRQOL indicators in relation to demographics (4,20), health risks (5,10,21), or specific health conditions (6-9,12,22). We dichotomized some predictors for the analysis (ie, sex, current smoking, alcohol use, physical activity, asthma, diabetes, obesity, and disability), whereas other predictors had multiple categories (ie, age, race/Hispanic ethnicity, income, and employment status). The definitions of the 12 predictors are available in our previous article (1). Reference groups chosen for the IRT model were those having the lowest risk for poor or fair general health and usually the lowest risk for the other HRQOL variables as well.

### 2-parameter dichotomous IRT model

We provide a basic description of IRT and present only essential mathematic formulas. Several sources provide more technical details (23-28). IRT, also known as latent trait theory, comprises a set of generalized linear models (27). IRT models are mathematical equations describing the association between a respondent's level for a latent trait, which is not measurable directly, and the probability of a particular item response using a nonlinear monotonic function (27). The latent trait we studied is HRQOL.

The figure shows the IRT model for the latent trait HRQOL with predictors and indicators. It includes 2 components. The relationship between the predictors and the latent trait is the structural component of the model. The relationship between the indicators and the latent trait is the measurement component of the model.

IRT now contains a large family of models. The simplest model is the Rasch (1960) model, which is also known as the 1-parameter logistic model (25). Popular unidimensional IRT models for dichotomous response data are the 1-, 2-, and 3-parameter logistic models (26). For each indicator, we used the 2-parameter dichotomous IRT model equation in equation no. 1:

Equation 1

Logit(Pi(k)∣θi)=α(k)(θi−β(k))

(k = 1, 2, 3, …… ,9)

The Greek letter α is the indicator discrimination parameter, β is the indicator difficulty parameter, k represents the indicator (outcome), and θ is the latent trait (HRQOL level), which can be calculated by equation no. 2.

Equation 2

θi=ui+c1.age+c2.sex+...+c12.disability

If we substitute equation no. 2 into equation no. 1, we have equation no. 3.

Equation 3

Logit(Pi(k)∣θi)=α(k).ui+(α(k).c1).age+(α(k).c2).sex+...+(α(k).c12).disability−α(k).β(k)

If $\alpha^{\left(k\right)}.u_{i}=u_{i}^{\left(k\right)}$,$\alpha^{\left(k\right)}.c_{1}=\beta_{1}^{\left(k\right)}$ , $\alpha^{\left(k\right)}.c_{2}=\beta_{2}^{\left(k\right)}$, …,$\alpha^{\left(k\right)}.c_{12}=\beta_{12}^{\left(k\right)}$ , and ${-\alpha }^{\left(k\right)}.\beta^{\left(k\right)}=\beta_{0}^{\left(k\right)}$, then equation no. 1 can be simplified as equation no. 4:

Equation 4

Logit(Pi(k)∣θi)=β0(k)+ui(k)+β1(k).age+β2(k).sex+.....+β12(k).disability

Equation no. 4 is a random intercept logistic model. In the typical application of IRT, marginal maximum likelihood estimation is used to calibrate the indicator parameters, and a normal distribution of respondent latent-trait scores is assumed (25).

Various software products can be used to analyze health outcomes data with IRT methods, including BIGSTEPS/WINSTEPS, MULTILOG, PARSCALE, and SAS. We used the SAS PROC NLMIXED procedure (SAS Institute Inc, Cary, North Carolina) to perform the IRT analysis. The *t* test was used to identify significant relationships (*P* [two-sided] < .05). SAS codes appear in the Appendix. The dataset was reorganized to have 1 row for each indicator. Therefore, a subject could have up to 9 rows, and subjects missing some indicators would have fewer rows. IRT analysis is not affected by missing data; that is, the IRT analysis was still viable using PROC NLMIXED even with incomplete data for the 9 indicators. This analysis is valid under the assumption of missing at random (29).

## Results

Overall, 14.8% had poor or fair general health; 28.8% reported lack of energy, and 23.8% reported inadequate sleep or rest (Table 1).

Table 2 highlights the performance of the 9 HRQOL indicators by displaying the values of α (indicator discrimination parameter) and β (indicator difficulty parameter) for each of the indicators. For each of the 9 indicators, α is statistically significant, meaning each indicator is able to discriminate reliably between good and poor for 1 aspect of HRQOL. The larger the value of β for each indicator, the higher the probability that the Rhode Island population has a poor HRQOL as measured by that indicator.

Table 3 displays how HRQOL (θ, latent variable) is related to the 12 predictors that are 0-1 variables, with 0 referring to the reference level. Interpreting these results is the same as if we were interpreting the results of a linear regression. Women have poor HRQOL (θ) compared with men, and the difference is significant. Poor HRQOL (θ) increased with decreasing levels of annual household income, and the differences are significant. Respondents who were unable to work or who were unemployed had significantly worse HRQOL (θ) than people in other employment categories. Homemakers/students and retired people had HRQOL (θ) similar to that of employed people. Current smokers, chronic alcohol users, or people who were physically inactive all had significantly worse HRQOL (θ) than nonsmokers, people who were not chronic users of alcohol, or who were physically active. People who had been told by a physician that they had diabetes or asthma were significantly more likely to have poor HRQOL (θ) than were people without these conditions. Obese people and disabled people were also more likely to have poor HRQOL (θ) than were nonobese or nondisabled people, and differences were significant. There were no significant differences for age or race/ethnicity groups.

## Discussion

IRT is a special type of structural equation model that has been applied in educational measurement with great success (24). In recent years, IRT methods have been used to develop measurement tools for health status assessment, for example, to construct instruments, score scales, or validate tests. These applications have focused on the measurement component of IRT. However, we have used IRT to integrate the analysis of multiple correlated outcomes. We focused on the structural component of the model (Figure), which characterizes the relationship between HRQOL (θ), demographics, risk factors, and health conditions.

We used an IRT model to analyze the BRFSS HRQOL data for 2 reasons. First, when we used multivariable logistic regression models to analyze the multiple correlated indicators in our previous study (1), each individual indicator (outcome) for HRQOL reflected only a specific aspect of physical health or mental health or both. The results of these multiple discrete models for HRQOL, which overlapped each another, were redundant and cumbersome to integrate into an overall evaluation. Finding a method to integrate these multiple correlated indicators into an encompassing simple indicator was our objective. IRT enabled assessment of overall HRQOL as an underlying or latent variable not amenable to direct measurement. It allowed evaluation of HRQOL (θ) in relation to demographics, health risks, and health conditions. Second, if any single indicator is used to assess HRQOL, its reliability can be compromised by the various factors that might influence an individual's response to any single indicator measure. If all indicators are considered together, the effect of this kind of variation for any single measure is reduced, improving the reliability of our assessment of HRQOL. IRT provides a solution to measuring HRQOL across multiple correlated indicators (outcomes).

Equation no. 2 represents the relationship between the latent trait and predictors, and equation no. 4 represents the relationship between indicators and predictors. In equation no. 4 for "Mentally unhealthy" in Table 2, α is 1.45; and in Table 3, the estimated coefficient for "Current smoker" is 0.27, thus OR = exp(α·c) = exp(1.45 × 0.27) = 1.5. In our previous analysis using a logistic regression model (1), the OR is also 1.5 for "mentally unhealthy" and "current smoker." Using this calculation, we can get similar results to those in our previous analysis, which was based on logistic regression models (1). This process illustrates how 1 IRT model can generate the results of 9 logistic regression models, and the results from the IRT model and the logistic regression models are similar. This also demonstrates that we can use 1 IRT model to combine results of multiple logistic regression models.

Our previous article (1) demonstrated that the prevalence of poor physical health increased with age, and the prevalence of poor mental health decreased with age. However, our IRT results indicate no significant difference in overall HRQOL (θ) between younger and older adults (Table 3). Our previous research (1) also showed that Hispanics had the highest percentage of "poor or fair" general health but did not have the highest percentage for other indicators of poor HRQOL. Research suggests that Hispanics who do not speak English fluently have lower educational achievement and lower levels of health literacy, which may make it difficult for them to respond to questions on HRQOL (30). Our IRT results show no difference in HRQOL (θ) among different racial/ethnic groups.

Results represented in Table 3 can enable health-related initiatives in Rhode Island to target specific populations at high risk for poor HRQOL (θ). Factors significantly associated with poor HRQOL (θ) were being female, having a household income less than $50,000, being unemployed or unable to work, being a smoker or chronic alcohol user, not engaging in leisure-time physical activity, having doctor-diagnosed asthma or diabetes, being obese, or having a disability.

Because IRT methods were originally developed for educational assessment with a homogeneous population (24,31), there is no guidebook that tells how to use IRT methods to evaluate health measures. There are many IRT models from which to choose, which means that finding a model that fits the available data and can estimate model parameters is difficult (24,26). IRT has the potential of being applied to other epidemiologic data with multiple correlated outcomes (32,33).

IRT methods may find increasing application in epidemiology. IRT may be a solution for modeling the multiple correlated outcomes often found in epidemiologic studies. This study provides a picture of the relation between overall HRQOL and demographics, behavioral risk factors, and health conditions. It indicates at-risk subpopulations in Rhode Island where interventions might have the most significant impact on HRQOL.

## Figures and Tables

**Table 1:** Selected Demographics, Risk Factors, Health Conditions, and Health-Related Quality of Life Indicators Among Rhode Island Adults (N = 3,999), Behavioral Risk Factor Surveillance System, 2004

**Demographics, Risk Factors, and Health Conditions^a^**	No. (Weighted %)^b^
**Age, y**	
18-44	1,524 (51.0)
45-64	1,588 (30.4)
≥65	837 (18.5)
**Sex**	
Male	1,531 (47.2)
Female	2,468 (52.8)
**Race/ethnicity**	
Non-Hispanic white	3,367 (84.7)
Hispanic	332 (8.8)
Other	244 (6.5)
**Annual income, $**	
<25,000	960 (24.9)
25,000-49,999	986 (28.2)
≥50,000	1,519 (46.9)
**Employment status**	
Unable to work	246 (4.7)
Unemployed	237 (6.0)
Homemaker/student	298 (10.3)
Retired	795 (17.3)
Employed	2,410 (61.7)
**Smoking**	
Current smoker	820 (21.3)
Not a current smoker	3,168 (78.7)
**Alcohol use**	
Chronic alcohol use	270 (7.6)
No chronic alcohol use	3,700 (92.4)
**Physical activity**	
Leisure time activity	1,026 (24.2)
No leisure time activity	2,971 (75.8)
**Asthma**	
Asthma	421 (9.6)
No asthma	3,559 (90.4)
**Diabetes**	
Diabetes	328 (7.2)
No diabetes	3,670 (92.8)
**Obesity**	
Obese (body mass index >30 kg/m^2^)	762 (19.0)
Not obese	3,016 (81.0)
**Disability**	
Have a disability	717 (15.3)
No disability	3,064 (84.7)
**Health-related quality of life indicator**	
Poor or fair general health	670 (14.8)
Activity limitation^c^	311 (6.8)
Physically unhealthy^c^	495 (10.6)
Pain-related activity limitation^c^	417 (9.7)
Lack of energy^c^	1,117 (28.8)
Mentally unhealthy^c^	455 (10.5)
Sad, blue, or depressed^c^	343 (8.2)
Worried, tense, or anxious^c^	516 (13.2)
Lack of rest or sleep^c^	879 (23.8)

a Variable descriptions are included in the Methods section and in Jiang et al (1).

b Data are reported as unweighted frequencies and weighted percentages.

c Respondents reported this indicator for ≥14 days/month.

**Table 2:** Estimated α and β Parameters Based on the 2-Parameter Item Response Theory Model, Rhode Island, Behavioral Risk Factor Surveillance System, 2004

Indicator^a^	Discrimination Parameter: α^b^ Estimate (95% CI)	Difficulty Parameter: β^b^ Estimate (95% CI)
Poor or fair general health	1.51 (1.34-1.69)	2.60 (2.41-2.80)
Activity limitation^c^	3.18 (2.57-3.80)	2.97 (2.77-3.17)
Physically unhealthy^c^	2.21 (1.91-2.50)	2.65 (2.47-2.84)
Pain-related activity limitation^c^	1.80 (1.57-2.04)	2.85 (2.65-3.06)
Lack of energy^c^	1.20 (1.06-1.33)	1.86 (1.68-2.04)
Mentally unhealthy^c^	1.45 (1.25-1.64)	3.11 (2.86-3.35)
Sad, blue, or depressed^c^	1.75 (1.50-2.01)	3.19 (2.95-3.42)
Worried, tense, or anxious^c^	1.29 (1.12-1.46)	2.98 (2.74-3.23)
Lack of rest or sleep^c^	0.69 (0.59-0.79)	2.84 (2.52-3.15)

Abbreviation: CI, confidence interval.

a Variable descriptions are included in the Methods section and in Jiang et al (1).

b Significant for all 9 indicators.

c Respondents reported this indicator for ≥14 days/month.

**Table 3:** Demographics, Risk Factors, and Health Conditions Regressed on Poor Health-Related Quality of Life (θ), Rhode Island, Behavioral Risk Factor Surveillance System, 2004

**Demographics, Risk Factors, and Health Conditions^a^**	Estimated Coefficients (95% CI)	*P* Value
**Age, y**		
18-44	1 [Reference]	NA
45-64	−0.06 (−0.17 to 0.08)	.46
≥65	−0.19 (−0.41 to 0.02)	.07
**Sex**		
Male	1 [Reference]	NA
Female	0.15 (0.04 to 0.26)	.006
**Race/ethnicity**		
Non-Hispanic white	1 [Reference]	NA
Hispanic	0.00 (−0.20 to 0.21)	.97
Other	0.05 (−0.16 to 0.26)	.65
**Annual income, $**		
≥50,000	1 [Reference]	NA
25,000-49,999	0.19 (0.06 to 0.33)	.004
<25,000	0.50 (0.35 to 0.65)	<.001
**Employment status**		
Employed	1 [Reference]	NA
Retired	0.03 (−0.17 to 0.23)	.79
Homemaker/student	0.04 (−0.18 to 0.25)	.74
Unemployed	0.56 (0.34 to 0.78)	<.001
Unable to work	0.83 (0.60 to 1.06)	<.001
**Current smoker**		
Not a current smoker	1 [Reference]	NA
Current smoker	0.27 (0.14 to 0.40)	<.001
**Alcohol use**		
No chronic alcohol use	1 [Reference]	NA
Chronic alcohol use	0.20 (0.00 to 0.40)	.04
**Physical activity**		
Leisure time activity	1 [Reference]	NA
No leisure time activity	0.44 (0.32 to 0.56)	<.001
**Asthma**		
No asthma	1 [Reference]	NA
Asthma	0.30 (0.14 to 0.46)	<.001
**Diabetes**		
No diabetes	1 [Reference]	NA
Diabetes	0.28 (0.10 to 0.46)	.002
**Obesity**		
Not obese	1 [Reference]	NA
Obese (body mass index >30 kg/m^2^)	0.22 (0.09 to 0.35)	<.001
**Disability**		
No disability	1 [Reference]	NA
Have disability	1.21 (1.07 to 1.35)	<.001

Abbreviations: CI, confidence interval; NA, not applicable.

a Variable descriptions are included in the Methods section and in Jiang et al (1).

## Appendix. SAS code for the item response theory analysis

proc nlmixed data=IRT;

parms b1=**2.6** b2=**3** b3=**2.7** b4=**2.9** b5=**1.9** b6=**3.1** b7=**3.2** b8=**3** b9=**2.8**

a1=**1** a2=**1** a3=**1** a4=**1** a5=**1** a6=**1** a7=**1** a8=**1** a9=**1**

c1=**0** c2=**0** c3=**0** c4=**0** c5=**0** c6=**0** c7=**0** c8=**0** c9=**0** c10=**0**

c11=**0** c12=**0** c13=**0** c14=**0** c15=**0** c16=**0** c17=**0** c18=**0**;

theta=c1*_agegrp1+c2*_agegrp2+c3*gender+c4*_race1+c5*_race2+

c6*_income1+c7*_income2+c8*_employ1+c9*_employ2+

c10*_employ3+c11*_employ4+c12*_rfsmok+c13*_rfdrhv+

c14*_actvity+c15*_asthma+c16*_diabets+c17*_obesity+

c18*_disblwo+u;

if indicator=**1** then eta = a1*(theta-b1);

else if indicator=**2** then eta = a2*(theta-b2);

else if indicator=**3** then eta = a3*(theta-b3);

else if indicator=**4** then eta = a4*(theta-b4);

else if indicator=**5** then eta = a5*(theta-b5);

else if indicator=**6** then eta = a6*(theta-b6);

else if indicator=**7** then eta = a7*(theta-b7);

else if indicator=**8** then eta = a8*(theta-b8);

else if indicator=**9** then eta = a9*(theta-b9);

p = exp(eta)/(**1**+exp(eta));

model Y ~ binary(p);

random u ~ normal(**0**, **1**) subject=id;

run;
